# Visual anticipation of the future path: Predictive gaze and steering

**DOI:** 10.1167/jov.21.8.25

**Published:** 2021-08-26

**Authors:** Samuel Tuhkanen, Jami Pekkanen, Richard M. Wilkie, Otto Lappi

**Affiliations:** 1 Cognitive Science, Traffic Research Unit, University of Helsinki, Helsinki, Finland; 2 Cognitive Science, University of Helsinki, Helsinki, Finland; 3 School of Psychology, University of Leeds, Leeds, UK

**Keywords:** eye movements, prediction, internal model, steering control, driving

## Abstract

Skillful behavior requires the anticipation of future action requirements. This is particularly true during high-speed locomotor steering where solely detecting and correcting current error is insufficient to produce smooth and accurate trajectories. Anticipating future steering requirements could be supported using “model-free” prospective signals from the scene ahead or might rely instead on model-based predictive control solutions. The present study generated conditions whereby the future steering trajectory was specified using a breadcrumb trail of waypoints, placed at regular intervals on the ground to create a predictable course (a repeated series of identical “S-bends”). The steering trajectories and gaze behavior relative to each waypoint were recorded for each participant (*N* = 16). To investigate the extent to which drivers predicted the location of future waypoints, “gaps” were included (20% of waypoints) whereby the next waypoint in the sequence did not appear. Gap location was varied relative to the S-bend inflection point to manipulate the chances that the next waypoint indicated a change in direction of the bend. Gaze patterns did indeed change according to gap location, suggesting that participants were sensitive to the underlying structure of the course and were predicting the future waypoint locations. The results demonstrate that gaze and steering both rely upon anticipation of the future path consistent with some form of internal model.

## Introduction

Anticipatory behaviors in humans can be observed in almost all skilled-action contexts, be it the timing of a ball catch or driving down a winding road at speed. The extent to which anticipation is driven by predictive internal models versus information directly available from the scene remains an open question.

Over the past 25 years, studies examining the control of steering have demonstrated that there is tight linkage between the information available from the environment, where drivers look (Land & Lee, 1994), and what kinds of eye movement strategies are used to retrieve that information (Lappi et al., 2020; for review, see Lappi, 2014; Lappi & Mole, 2018). It has also been shown experimentally that instructing people to keep to a particular lane position biases where they look, and having them adopt a specific gaze strategy biases the steering responses produced (Wilkie & Wann, 2003b; Kountouriotis et al., 2013; Mole et al., 2016), indicating that there is a natural coupling between steering and gaze. The information sampled via active gaze behaviors could be supplied by a number of features from across the visual field: Optic flow (from the apparent motion of textured surfaces; Gibson, 1986), retinal flow (optic flow altered by eye-movements; Cutting et al., 1992; Wilkie & Wann, 2003a; Matthis et al., 2021), and tangent points (Tau Lee, 1976; Raviv & Herman, 1991; Land & Lee, 1994) have all been analyzed as potential sources (for review, see Cutting, 1986; Regan & Gray, 2000; Wann & Land, 2000).

While a variety of sources of information have been identified across different environments, the precise relationship between the gaze behaviors exhibited (where you look and when) and the sampling of each source is still not fully understood. It has been shown that in many everyday locomotor contexts, such as driving (Lappi et al., 2013, 2020), bicycling Vansteenkiste et al., 2014), and walking (Grasso et al., 1998; Matthis et al., 2018), gaze appears to land on and track fixed “waypoints” that may (or may not) be specified by some visible marker. Recent evidence has demonstrated that the gaze behaviors produced when steering along a path defined using only a series of marked waypoints are comparable to those generated when steering along a winding road (Tuhkanen et al., 2019). Furthermore, when steering via visible waypoints, a regular gaze pattern akin to “optokinetic pursuit” occurred: looking to the next waypoint (∼2 s ahead), tracking this point with a pursuit eye movement (for ∼0.5 s) before generating a further “switch” saccade to look at the next waypoint in the sequence. Crucially, when the next waypoint in the sequence was not visible, gaze behavior followed a very similar pattern, suggesting that much of the gaze–pursuit–switch pattern was internally driven, rather than being solely driven by the external visual stimulus. The nature of the internal driver of visual guidance will be further examined in this article: Specifically, can visual sampling behavior be understood as a simple repeating “motor program,” or does it actually reflect genuinely predictive information processing? Answers to this fundamental question will lead to a better understanding of locomotor control, particularly the ability of humans to drive vehicles at speed.

### Aims of this study

In a previous study, we presented participants with a path specified by waypoints embedded in rich optic flow (Tuhkanen et al., 2019). Gaze spontaneously tracked these waypoints during the approach, with saccades being generated toward the next waypoint location further in the distance in an anticipatory manner (i.e., even when the next waypoint failed to appear in the expected location, participants made saccades to the approximate location of the “missing waypoint”). Through a careful analysis of the saccade characteristics, many online/heuristic strategies could be ruled out (i.e., gaze patterns were found not to be consistent with simple bottom-up visual transitions or generating saccades toward salient locations). These analyses suggested that for this phase of the locomotor task at least (traveling on a constant radius bend), the anticipation of future waypoints appeared to be genuinely predictive.

One question this evidence does not directly answer is: What happens when the road curvature is not constant but changes in a predictable manner? If the observer is driving along a path constructed from a repeated series of identical S-bends (with the same curvature), then the regularity and predictability of the scene may lead to stored representations about waypoint locations that inform gaze patterns. A coarse form of representation might lead the driver to predict a waypoint ahead, in the current direction of travel at regular intervals of time/space. But with repeated connected bends that are all equally long, the likelihood of a change in direction (i.e., from a leftward bend to a rightward bend or vice versa when steering through a repeating series of S-bends) increases the deeper one has traveled into each bend. Despite the previous research that highlights tight coupling between gaze and steering behaviors, there are a number of questions for which we still do not have clear answers: Can drivers produce saccades that reliably predict future waypoint locations even when the bend changes, direction? Will anticipatory gaze behaviors change depending on how deep the driver is into each bend? If so, will steering trajectories reflect gaze changes, or is it the case that predictive gaze behaviors start to become decoupled from steering control?

A strong focus of (Tuhkanen et al., 2019) was comparing gaze patterns between winding roads (demarcated by road lines) and paths specified only by waypoints. The structure of the scenes used meant that bends were quite long, and advance notice was given of changes in bend direction from visual feedback before each inflection point. The scene structure limited further analysis of what form of prior information may have been stored to aid predictions about the future path. In the present design, the bends used were shorter and the placement of the waypoints purposely created situations where it was sometimes impossible to anticipate upcoming changes in direction from visual feedback alone. As such, the present experimental design should elicit one of the following possible behaviors:

(A) Participants look only at visible waypoints (though this behavior is not expected since this was not observed previously by Tuhkanen et al. (2019).
(B) Participants look ahead to the predicted location of the “missing waypoint” based on the previous waypoint location (i.e., prediction of the future path is limited to constant-curvature bends).
(C) Participants look ahead to the predicted location of the “missing waypoint” based on some representation of the S-bend structure as well as an internal estimate of current (spatial or temporal) position.

## Method

**Figure 1.**
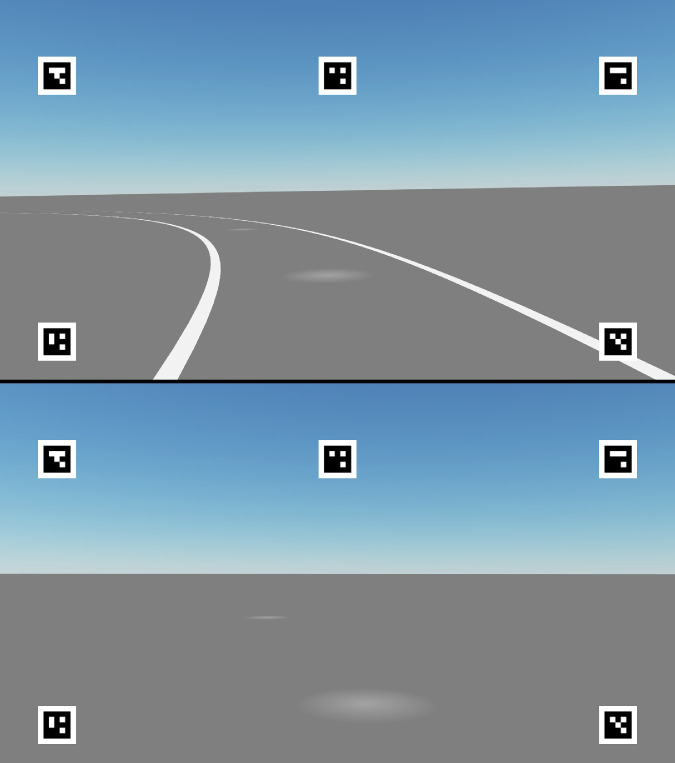
Stimuli. *Top panel*: Sample screenshot from a practice trial of the experiment where the track edges were visible. *Bottom panel*: Sample screenshot from a test trial of the experiment where the track edges were invisible. The track was specified by gray/white circular waypoints that were presented 80% of the time (VIS condition) at 1-s intervals and 20% of the time (MISS condition) at 2-s intervals with a virtual “missing waypoint” in between two visible ones. The waypoints always appeared at a 2-s time headway (TH) from the driver. The five square markers were fixed on the screen and used to determine a homography from the eye tracker's camera image to the screen.

Participants drove along a continuous winding course specified by a series of visible waypoints generated within a fixed-base driving simulator (open source, available at https://github.com/samtuhka/webtrajsim/tree/birch20). The course was displayed visually using intermittently appearing waypoints as shown in Figure 1 (to control the available preview information of the road ahead). Participants were asked to stay on the “track”—track edges were only indicated visually during the practice trial; during the experimental trials, an auditory tone indicated that the driver had deviated more than 1.75 m from the (invisible) track center. Steering was controlled using a Logitech G920 Driving Force (Logitech, Fremont, CA) gaming wheel (auto-centering was on but otherwise there was no force-feedback). The simulator used a raycast vehicle model (as implemented in the Cannon.js JavaSript physics library) to simulate vehicle physics. Locomotor speed was kept constant at 10.5 m/s (no gears, throttle, or brakes were used). Eye movements were recorded with a head-mounted eye tracker. The simulator ran at 60 Hz and was displayed on a 55–in. LG 55UF85 monitor. The experimental design was similar to the Experiment 2 described in Tuhkanen et al. (2019), with the main differences being the waypoint placement and design in order to better gauge how participants anticipate and react to the change in direction as they near and pass the inflection point of an S-bend. In the previous experiment (Tuhkanen et al., 2019), the bends were longer, meaning there were less data from inflection points, and the change in direction was easier for participants to discern as there was always at least one visible waypoint before the inflection point from which it was possible to determine whether the bend was about to change direction.

### Participants

A sample of 16 participants (8 female, 8 male, mean age = 30, *SD* = 7, range = 22–47) was recruited through University of Helsinki mailing lists. All participants had a minimum of 20,000 km of driving experience and reported normal or corrected vision. After the experiment, the participants were rewarded with three exercise and cultural vouchers worth €15 in total for participation.

### Eye tracker

The eye movements of the participants were recorded with Pupil Core (Pupil Labs UG haftungsbeschränkt, Berlin, Germany) eye tracker. Binocular cameras recorded at 60 Hz at 640 × 480 resolution while one forward-camera recorded the scene at 30 Hz at 1,280 × 720 resolution. The open-source Pupil Capture software (https://github.com/pupil-labs/pupil) was used to record and calibrate the eye tracker. No head or chin rest was used, and participants were given no gaze instructions outside the calibration.

The eye tracker was calibrated at the beginning of the experiment and between every few trials (generally, every two trials at the experimenter's determination). The “2D pipeline” of Pupil Capture was used for calibration (i.e., a polynomial fit between the center positions of the pupil and the detected marker positions was used to estimate gaze position). The gaze signals from both eye cameras were averaged together to a cyclopean gaze signal. In Tuhkanen et al. (2019), we estimated the mean calibration accuracy when using a nearly identical procedure to be approximately 1 degree. Mean accuracy here refers to the mean angular distance between the calibration marker centers and gaze as measured at the end of the trials in Tuhkanen et al. (2019).

### Gaze signal segmentation and classification

The gaze signal of the participants was mapped to the coordinates of the screen by determining a homography from the camera coordinates with the aid of five square optical markers (see Figure 1). See Movie 4 for a sample on what the resulting transformation from the eye tracker's forward camera to screen coordinates looks like. The pixel coordinates were then transformed to (a linear approximation of) angular coordinates. The horizontal field of view (FoV) rendered was 70${}^{\circ }$, with the fixed observer viewing distance (0.85 m) matched to this rendered FoV. All gaze points were included in the data with no additional filtering. Saccade detection and all analyses use the gaze-on-screen position signal.

The gaze signal was classified with the naive segmented linear regression (NSLR) algorithm (Pekkanen & Lappi, 2017) into individual saccades, fixations, and smooth-pursuit segments. The algorithm approximates a maximum likelihood linear segmentation – (i.e., the gaze data are modeled as successive linear segments). The accompanying hidden Markov model (NSLR-HMM) classifies the different segments into saccades, fixations, smooth pursuits, and postsaccadic oscillations. In our experience, the saccade identification is the most reliable component and was the only one used in our analyses.

### Design and stimuli

**Figure 2.**
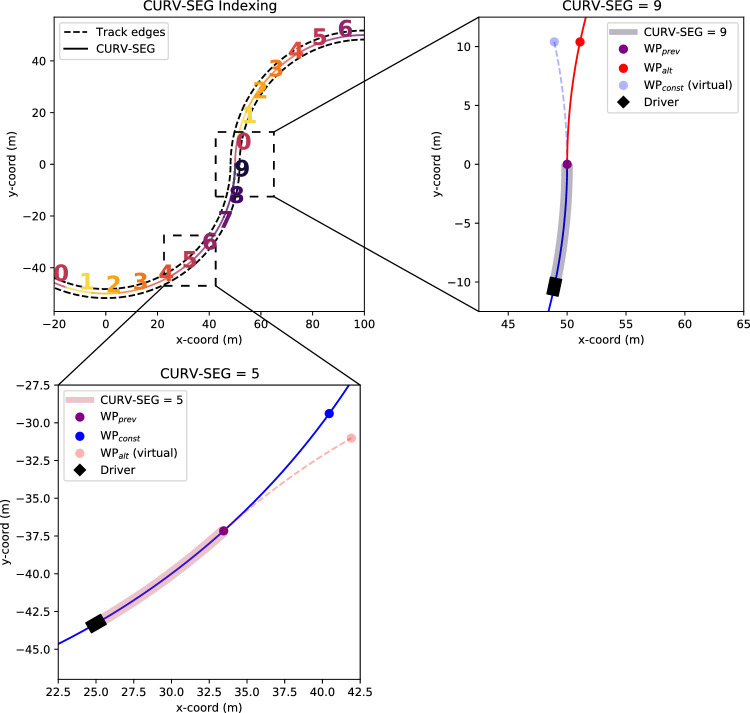
Bird's-eye view of the geometry of the track and the indexing convention of the curve segments. The participants drove through 50-m radius curves alternating to left and right. Each curve consisted of 10 (visible or missing) waypoints and segments. *Top left panel*: Indexing of the curve segments (CURV-SEG) used for analysis purposes to divide the data. CURV-SEG = 0 starts when the driver passes through the inflection point between two curves. Note only waypoints, not track edges, were visible during experimental trials *Top right panel*: WP placement during CURV-SEG = 9—the final CURV-SEG before the inflection point. The driver (black rectangle) is set at the beginning of a CURV-SEG while the dots indicate the positions of WP_const_ (blue), WP_prev_ (purple), and WP_alt_ (red). The red trajectory indicates the true centerline following the inflection point and WP_alt_ the location of the most distant WP while the dashed blue trajectory and WP_const_ (virtual) indicate where the path would continue assuming a constant-curvature path. *Top right panel*: WP placement during CURV-SEG = 5. The constant-curvature assumption is correct here, and always when CURV-SEG ≠ 9.

The participants drove through a track consisting of 120${}^{\circ }$ arc curves (radius = 50 m) alternating to left and right (see Figure 2). A single trial consisted of 16 constant-radius curves and took approximately 160 s to complete. The track width was 3.5 m, though the track edges were only visible in the first practice trial. If the participant drove off the track (absolute track position > 1.75 m from the centerline), the simulator started playing a constant “beeping” sound until the participant returned to the track.

The participants completed three practice trials at the start of the experiment (practice trials were shorter than test trials and lasted for approximately 50 s). In the first practice trial, the edge lines of the track were visible to introduce the participants to the track and get familiar with the dynamics of the virtual car. A sample of the first practice trial can be seen in Movie 1. In the second practice trial, the track could only be discerned through the waypoints, but unlike in the test trials, there were no missing waypoints. See Movie 2 for a sample trial of the second practice. The third practice trial was identical to the test trials. The actual experiment consisted of 10 test trials. See Movies 3–4 for sample test trials. In addition, there were six randomly placed control trials where there were no missing waypoints, but these were ultimately not utilized in the analysis.

The speed of the virtual vehicle was kept constant at approximately 10.5 m/s, corresponding to a 12 ${}^{\circ }$/s yaw rate during constant-curvature cornering. The virtual camera was at the height of approximately 1.2 m from the ground. The ground had a solid gray texture (producing zero optic flow)—this was to keep the experimental design as similar as possible to Tuhkanen et al. (2019) and to ensure that the waypoints were the only visual cues of the driver's position. In test trials, the track could visually be discerned only through waypoints (in addition to the apparent movement of waypoints, vehicle roll provided another self-motion cue).

The rendered waypoints were 0.7 m radius circles/“blobs” with a linearly fading white texture (see Figure 1). The waypoints were not visible at all distances but rather appeared when they were at a 2-s time headway (TH, as defined by a point ahead along the centerline of the track from the driver at the fixed locomotor speed). The waypoints were normally (VIS condition) equally placed at a distance of approximately 10.5 m from one another, corresponding to 1 s in travel time. However, as a manipulation, approximately 20% of the waypoints did not appear (MISS condition). These “missing waypoints” were always separated from one another by at least three visible waypoints. When comparing the VIS condition to the MISS condition, to make the conditions as comparable as possible with the only exception being the visibility of the furthest waypoint (WP), only segments where the last three waypoints had been visible were included in the analysis of the VIS condition.

In total, each constant-radius curve consisted of 10 waypoints (see Figure 2). As the waypoints were located at the same angular positions in every curve, we discretized and indexed each curve into 10 segments (referred to from here on as CURV-SEG) with the index changing when the furthest WP changes. Right and left turning curves were considered equivalent, with left-turning curves being mirrored for all analyses.

CURV-SEG refers to the sections of the track where the furthest waypoint, whether visible or not, is at 1 s < TH ≤ 2 s. The driver passes the inflection point between two curves when they enter CURV-SEG = 0. At CURV-SEG = 8, the most distant WP is at the inflection point. The most distant WP at CURV-SEG = 9 is the first waypoint in the opposite direction to the current curve. In other words, it is the first of the waypoints that are located on the upcoming curve (the first waypoint after the inflection point). Even though they were not visible, missing waypoints in the MISS condition were treated the same way in the indexing scheme.

Waypoints were further classified as WP_const_ or WP_alt_ on the basis of whether they were on the constant-radius curve or not. When CURV-SEG ≤ 8, WP_const_ is the most distant WP (visible or missing). At CURV-SEG = 9, however, WP_alt_ is the most distant WP (visible or missing).

We still determined a WP_alt_ for every CURV-SEG—these served as virtual waypoints “where the next waypoint should appear if the previous waypoint was in fact an inflection point.” This was to probe whether participants would direct gaze according to this “alternative hypothesis,” especially in the MISS condition (see Figure 2). Similarly, we determined a virtual WP_const_ for CURV-SEG = 9 (“where the next waypoint should appear if the last waypoint was in fact not an inflection point”). We use the convention WP_prev_ to refer to the waypoint at 0 < TH ≤ 1 (i.e., the waypoint that had appeared at TH 2 s at the start of the previous CURV-SEG).

**Figure 3.**
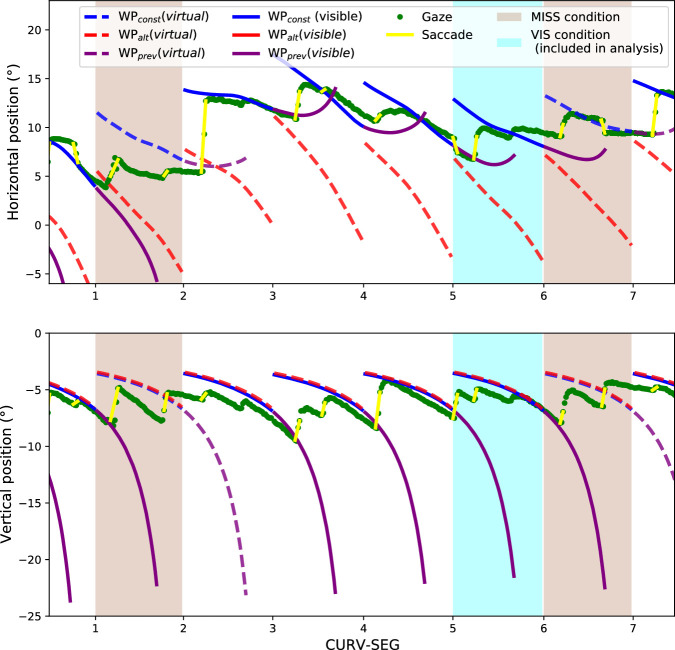
A sample gaze and waypoint time series from a single participant. *Top panel*: Horizontal screen positions in respect to CURV-SEG. Gaze tracking is depicted as green points while the yellow lines indicate the saccades that were derived using the NSLR-HMM algorithm. Blue lines indicate the location of WP_const_, red the location of WP_alt_, and purple the location of WP_prev_. Solid lines indicate the WP in question is visible, whereas dashed lines indicate that it is missing/virtual. WP_const_ is always visible except when CURV-SEG = 9 and the MISS condition does not apply. WP_alt_ is only visible when CURV-SEG = 9 and the VIS condition applies. WP_prev_ is visible when the previous WP_const_ (CURV-SEG ≠ 9) or WP_alt_ (CURV-SEG = 9) is visible. In the sample times series, the sienna-colored sections highlight the MISS manipulations and the cyan-colored sections highlight the part of the VIS condition that was included in the data analysis (i.e., when the furthest WP is visible and the last three WPs have all been visible). *Bottom panel*: Same as top panel but with vertical positions depicted instead.

A sample time series of the screen positions of the WPs and the gaze signal can be seen in Figure 3.

### Steering and overall performance

We did a cursory analysis of steering in the VIS and MISS conditions in respect to CURV-SEG. This was done in order to examine whether the conditions were comparable to one another and whether the different CURV-SEGS were comparable to one another. The reasoning was to minimize the possibility that any differences in gaze behavior between the conditions and with respect to CURV-SEG could not simply be explained differences in steering.

**Figure A1.**
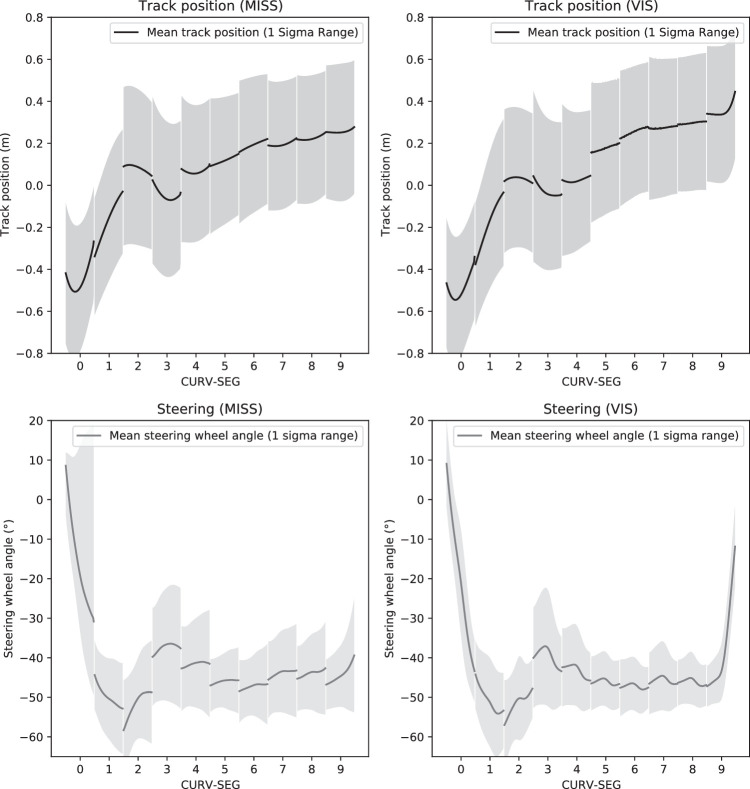
*Top left panel*: Mean track position and standard deviation pooled from all participants in the MISS condition in respect to CURV-SEG. Positive values indicate the position is more toward the outer edge. *Lower left panel*: Corresponding mean track position and standard deviation in the VIS condition. *Top right panel*: Mean steering wheel position and standard deviation pooled from all participants in the MISS condition in respect to CURV-SEG. Negative values indicate the steering wheel is in the direction of the curve. *Lower right panel*: Corresponding steering wheel position and standard deviation in the VIS condition.

Mean track position (i.e., distance from centerline) and mean steering wheel angle in the different conditions are shown in Figure A1. Steering behavior during CURV-SEG = 0 (where there is a transition to change bend direction) differs from the other segments where steering remains relatively stable. To avoid confounds related to differential steering for VIS and MISS conditions, CURV-SEG = 0 was excluded from further analyses.

In terms of overall steering performance, the participants drove off the track relatively rarely. An auditory tone was sounded to give feedback to the participant that they had left the track. The median participant spent approximately 1% of the total runtime off the track (range = 0.1%–4.0%). Approximately 99% of the total off-track time happened during the VIS condition—presumably following a recent missing waypoint (each missing waypoint was always followed by a minimum of three visible waypoints).

## Results

**Figure 4.**
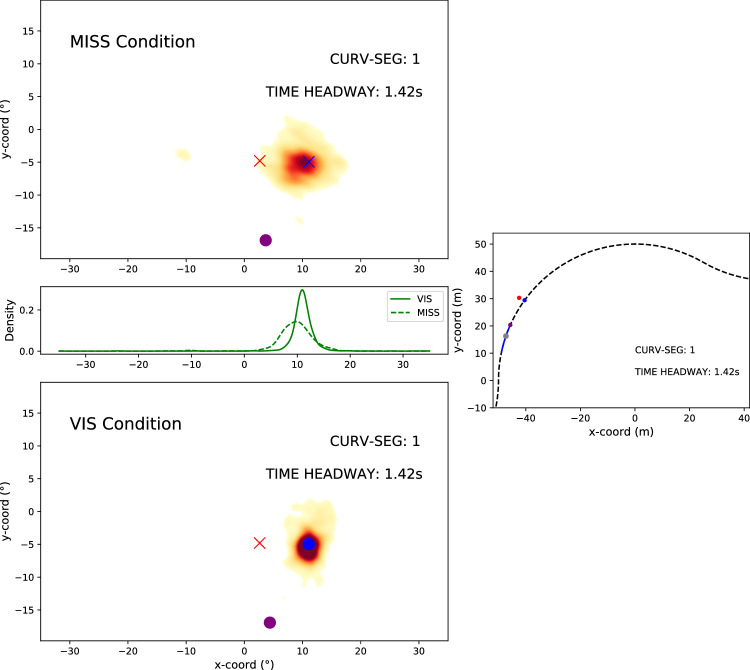
Sample frame from Movie 5. *Top panel*: The gaze density distribution (across all participants and trials) in the MISS condition in CURV-SEG = 1, at the time point when the time headway to the furthest (but missing) waypoint is 1.42 s. The blue cross indicates the position of the (missing) WP_const_, the red cross the virtual WP_alt_, and the purple dot WP_prev_ (i.e., the last waypoint that was actually observed). All the coordinates have been normalized with respect to the position of WP_const_, and the relative positions between WP_alt_ and WP_const_ should be effectively constant across trials. (Due to projection geometry, it changes with time headway.) The full movie shows the gaze distribution developing across each CURV-SEG. All left-turning segments of the data have been mirrored. *Middle left panel*: The marginal gaze distributions for the x-axis in the VIS and MISS conditions. *Middle right panel*: Bird's-eye view of the track. The black dotted line indicates the (invisible) centerline of the track and the solid blue line the extent of the CURV-SEG 01. The blue dot indicates the location of WP_const_, the red dot the location of WP_alt_, and the purple dot the location of WP_prev_. The gray dot indicates the position of the driver within the CURV-SEG. *Bottom panel*: The comparable gaze density distribution as in the top panel but in the VIS condition where the waypoint at TH = 1.42 s is, in fact, visible (having appeared at TH = 2.0 s). The blue dot indicates the location of the visible WP_const_ and the red cross the WP_alt_.

The general pattern of the participant gaze distribution at each point in time and each CURV-SEG is visualized in Movie 5 (https://doi.org/10.6084/m9.figshare.14828928, see Figure 4 for a sample frame). As in Tuhkanen et al. (2019), participants shift their gaze from the previous waypoint to both the visible and missing waypoint locations ahead. Unlike in Tuhkanen et al. (2019), however, in the case of the MISS condition, the gaze distribution appears to horizontally spread out more as CURV-SEG increases; this possibly reflects increased uncertainty over the location of the future path/waypoint and whether it continues along the current constant-curvature path. To investigate the robustness of this effect and its implications, three main analyses were performed followed by a report on participant strategies. First, we investigated whether the horizontal shift in gaze was significant within participants by looking at horizontal gaze offset from a reference point midway between WP_const_ and WP_alt_. Second, we investigated whether the effect persisted when restricting the analysis to examine the saccade landing points. Third, we investigated whether the anticipatory (or seemingly anticipatory) gaze fixations in the vicinity of WP_alt_ correlated with changes in steering. Finally, we discuss the survey of participant strategies.

### Gaze offset

If gaze and steering control employ purely online mechanisms (i.e., mechanisms driven by directly available external stimuli), there is little apparent reason to expect gaze behavior to change as a function of how far along a constant-radius curve participants have traveled (i.e., similar behavior for CURV-SEG = 1 and CURV-SEG = 9), especially since the visual scene is very similar. In contrast, predictive strategies (whether truly model based or not) should lead to greater uncertainty about whether future WPs will continue to fall along the same constant-radius arc as CURV-SEG increases. If this uncertainty is also reflected in gaze behavior, you should see more gaze polling at the vicinity of WP_alt_ and/or hedging somewhere between WP_alt_ and WP_const_. (Although if the predictive ability were perfect, the change in behavior from WP_const_ to WP_alt_ might only happen at CURV-SEG = 9, when the waypoint actually appears, or fails to appear, at the WP_alt_ location.)

To investigate this, we looked at how and if participants’ median horizontal gaze position (in the region horizontally spanning from WP_const_ to WP_alt_) would shift as a function of CURV-SEG. For each participant and each CURV-SEG, we determined a median horizontal gaze offset from the horizontal middle point between WP_const_ and WP_alt_—positive values indicate the gaze was closer to WP_alt_ than WP_const_.

Only the last frame of each CURV-SEG was included (i.e., the moments in time when the appearance of the next WP was most imminent). This exclusion was done to filter out gaze points that may have still been located in the vicinity of WP_prev_. As can be seen in Movie 4, for the last frames of a CURV-SEG, the participant gaze is vertically situated mostly at the height (screen y coordinates) of WP_const_ and WP_alt_. In addition, CURV-SEG = 0 was excluded from the analysis due to incomparable steering behavior and because it might still be ambiguous to the driver whether the inflection of the curve has in fact occurred (i.e., whether target path curvature has changed from right-hand to left-hand or vice versa).

**Figure 5.**
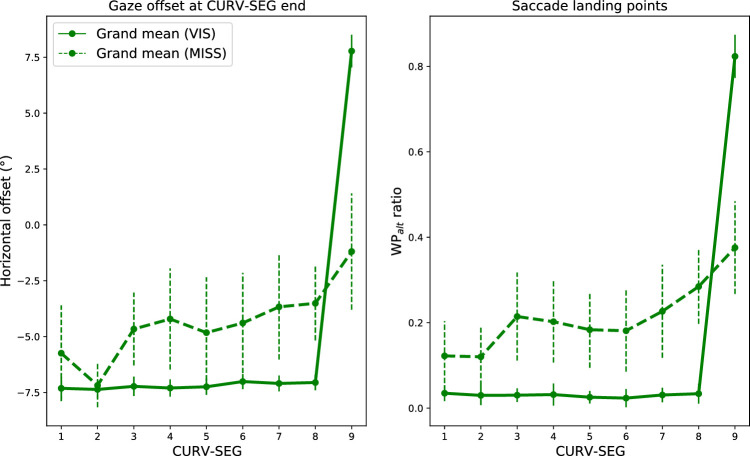
*Left panel*: The grand mean horizontal gaze offsets in the VIS (solid green line) and MISS (dashed green line) conditions. The offsets have been determined as the angular offset from the reference point in the middle between the WP_const_ and WP_alt_. Positive values indicate that the participants' gaze is on average horizontally closer to WP_alt_ than WP_const_. The grand mean was determined by calculating the mean from participant-wise median offsets. The error bars display the 95% CIs. In the VIS condition, the mean gaze position stays uniformly at the approximate location of WP_const_ (–6 degrees) when CURV-SEG ≤ 8 and shifts to the approximate position of WP_alt_ (6 degrees) at CURV-SEG = 9. In contrast, in the MISS condition, the mean gaze position gradually shifts toward WP_alt_ (though still remains closer to WP_const_ than WP_alt_) as CURV-SEG increases. *Right panel*: The mean ratio of saccades classified as landing toward WP_alt_ in the VIS and MISS conditions in respect to CURV-SEG. Before averaging the participants, each saccade was classified in respect to which waypoint (WP_alt_, WP_const_, WP_prev_, or MID) was nearest to the saccade landing point, and the proportion of saccades landing toward WP_alt_ was determined for each participant (which were then averaged). In the VIS condition, almost none of the saccades land toward WP_alt_ except when CURV-SEG = 9 when almost all do (this is expected as in the VIS condition, WP_alt_ is visible when CURV-SEG = 9, whereas WP_const_ is at all other times). In the MISS condition, the ratio gradually increases as CURV-SEG increases, possibly indicating greater uncertainty on whether the track will continue along the same constant-radius arc.

**Figure A2.**
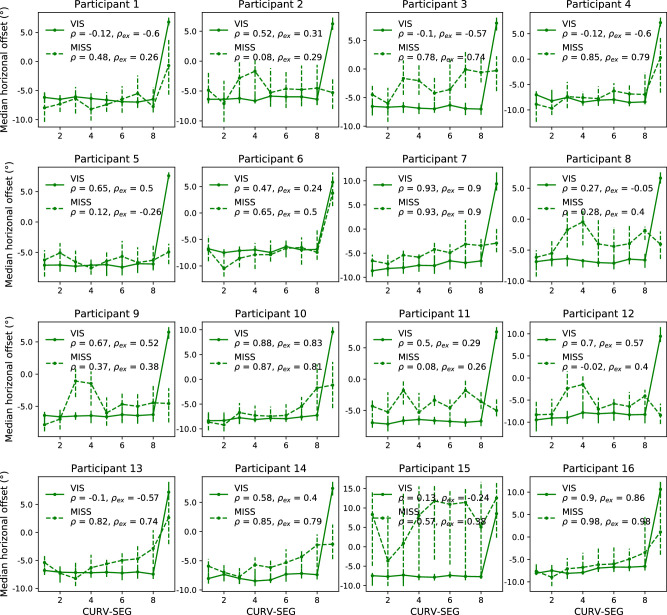
Horizontal gaze offsets for each participant in the VIS (solid green line) and MISS (dashed green line) conditions. The offsets have been determined as the median angular offset from the reference point in the middle between the WP_cont_ and WP_alt_. Positive values indicate that the participant's gaze is on average horizontally closer to WP_alt_ than WP_const_. ρ indicates Spearman's rho when 1 ≤ CURV-SEG ≤ 9 are all included and $\rho_{ex}$ Spearman's rho when CURV-SEG = 9 is excluded. Error bars display the 25th–75th percentile range.

Mean gaze offsets can be seen in Figure 5 in regard to both the MISS and VIS conditions. Participant-wise median offsets can be seen in Figure A2. For most participants, the median offset appears to increase as CURV-SEG increases. To assess this quantitatively, Spearman's rank correlation coefficient was used to estimate the correlation between CURV-SEG and the median offsets.

After a Fisher's *z* transformation, we derived a (retransformed) mean correlation coefficient of 0.67 for the MISS condition, with all but one participant having a positive correlation. The Fisher-transformed correlations were significantly different from zero (one-sample *t* test, *p* = 0.0002), indicating that the participants’ gaze drifted more toward the direction of WP_alt_ the further along a constant-radius curve the participants were.

If CURV-SEG = 9 is excluded from the data, the mean Spearman's rho is 0.62 with all but one participant having a positive correlation (one-sample *t* test to test difference from zero, *p* = 0.0002). Conversely, in the VIS condition when CURV-SEG = 9 is excluded, the mean Spearman's rho is 0.25, with 10 participants having a positive correlation (dependent *t* test to test difference from the MISS condition, *p* = 0.02) —that is, with this exclusion, participants on average had a lower Spearman's rho in the VIS condition than in the MISS condition.

### Saccade landing point classification

The results of the offset gaze analysis described in the previous section could, in principle, be explained by a decrease in generating saccades toward WP_const_ without a concomitant increase in looking toward WP_alt_. Another way to investigate the underlying representation used by participants is to see whether more saccades target WP_alt_ in the MISS condition the deeper into each constant-radius bend the participant has traveled (i.e., as CURV-SEG increases). The motivation of this investigation was to determine whether the participants would actively poll the alternate waypoint location (i.e., toward a predicted upcoming change in bend direction) the deeper they were into the bend.

In addition to using WP_const_, WP_alt_, and WP_prev_ for the saccade classification analysis, a MID classification was also created. The location of MID was determined as the arithmetic mean of the screen positions of WP_prev_, WP_alt_, and WP_const_. The purpose of this gaze category was to help classify gaze behavior if participants do not (clearly) target any of the waypoint locations and instead place their gaze somewhere between the alternatives locations (potentially in order to be able to detect WPs in either alternative location in the visual periphery).

**Figure A3.**
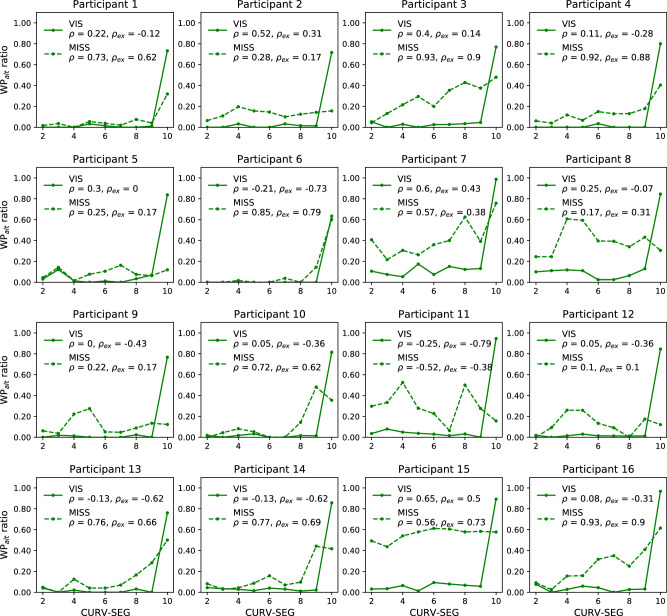
Ratio of saccades classified as landing toward WP_alt_ in the VIS (solid green line) and MISS (dashed green line) conditions. Each participant and the Spearman's rho between CURV-SEG and the WP_alt_ ratio for the participant in question are displayed separately. ρ indicates Spearman's rho when 1 ≤ CURV-SEG ≤ 9 are all included and $\rho_{ex}$ Spearman's rho when CURV-SEG = 9 is excluded.

This analysis determined the distance (in screen coordinates) between each saccade landing point to WP_alt_, WP_const_, MID, and WP_prev_. Each saccade landing point was then classified (as one of the three WPs or MID) depending on which region it was closest to. For each participant, the ratio of saccades in the WP_alt_ with respect to CURV-SEG was calculated (CURV-SEG = 0 was excluded as described in the previous analysis). The ratios with respect to CURV-SEG are visualized in Figure 5. Participant-wise ratios can be seen in Figure A3. As in the offset analysis, Spearman’ *r* was used to measure the correlation between CURV-SEG and the ratio of WP_alt_ classified saccades.

After a Fisher's *z* transformation, we derived a (retransformed) mean correlation coefficient of 0.63 for the MISS condition with all but one participant having a positive correlation. The *z*-transformed correlations were significantly different from zero (one-sample *t* test, *p* = 0.0003) indicating that the participants’ saccades landed more often in the vicinity of WP_alt_ as CURV-SEG increased (and the inflection point of the bend was approached).

If CURV-SEG = 9 is excluded from the data, the mean Spearman's rho is 0.57 with all but one participant having a positive correlation (one-sample *t* test to test difference from zero, *p* = 0.0003). Conversely, in the VIS condition when CURV-SEG = 9 is excluded, the mean Spearman's rho is −0.24 with only four participants having a positive correlation (dependent *t* test to test difference from the MISS condition, *p* = 0.0001) —that is, with this exclusion, participants on average had a lower Spearman's rho in the VIS condition than in the MISS condition.

### The effect of gaze position on steering

We have observed that gazing behavior shifts toward WP_alt_ in the MISS condition as CURV-SEG increases. To see if this at least seemingly predictive gaze behavior has any effect on steering, we investigated the correlation between gaze position near the inflection point (e.g., at the end of CURG-SEG = 9) at each run and what their future trajectory was.

**Figure 6.**
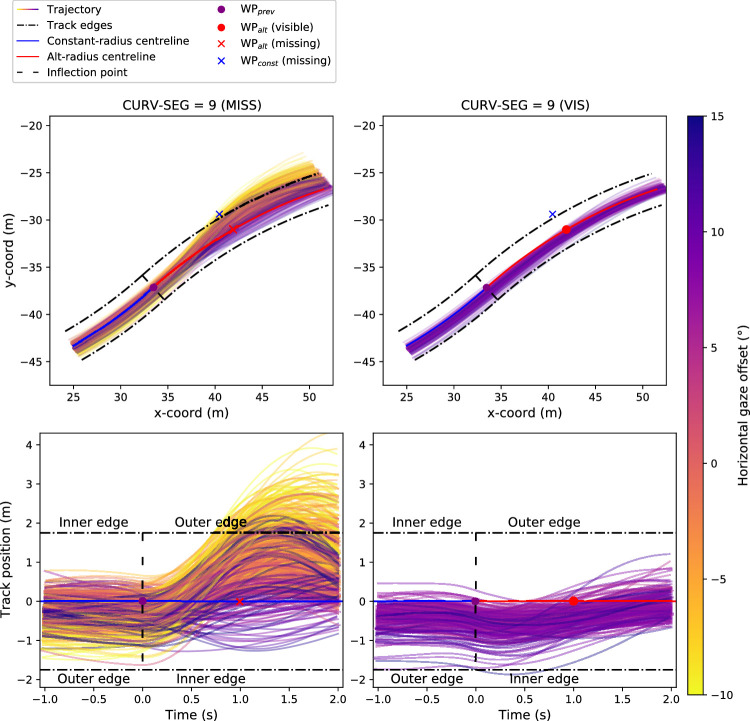
*Top left panel*: Participant trajectories in regards to track positions from −1 s before the inflection point to +2 s after the inflection point when WP_alt_ during CURV-SEG = 9 in the MISS condition. The individual trajectories have been colored by the horizontal gaze offset at the end of CURV-SEG = 9. Positive gaze offset indicates the gaze was closer to WP_alt_ than WP_const_ and negative that gaze was closer to WP_const_. When gaze offset is more toward WP_const_, the participants appear to steer closer toward the outer edge as opposed to steering closer toward the inner edge when gaze is closer to WP_alt_. *Top right panel*: Comparable to the top left panel but from the VIS condition. Only trajectories where WPs are visible during the whole –1- to +2-s duration (i.e., the two following CURV-SEGs are also part of the VIS condition). *Lower left panel*: Participant trajectories in regards to x,y coordinates from –1 s before the inflection point to +2 s after the inflection point when the furthest waypoint during CURV-SEG = 9 was missing. Note that the track edges and centerline were not visible (leaving the track caused a warning beep to sound). *Lower right panel*: Comparable to the lower left panel but from the VIS condition. Only trajectories where WPs are visible during the whole –1- to +2-s duration (i.e., the two following CURV-SEGs are also part of the VIS condition).

**Figure 7.**
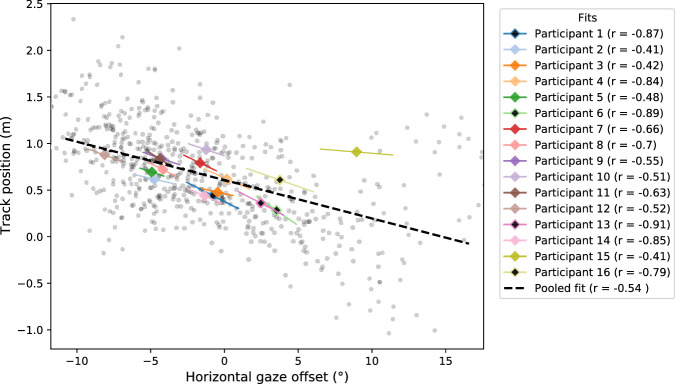
Scatterplot of track position as a function of horizontal gaze offset at the end of CURV-SEG = 9. Small gray dots indicate individual runs from all participants when WP_alt_ was missing during CURV-SEG = 9. Mean track positions have been calculated from the 2 s following the inflection point. Colored lines indicate the slopes of participant-wise linear OLS fits (diamond the location of the participant mean)—line width is proportional to participant's standard deviation of gaze offset (total width = 0.5 * *SD*). Participants 1, 6, 13, and 16 reported to have counted the WPs (see participant strategies) and are marked with a black center within the diamond marker. The pooled fit (dashed black line) has been calculated from the data of all participants (note that the *r* is different from the mean participant *r* of –0.7). All participants have a negative correlation, and the Participant means except for Participant 15 appear to mostly follow the general trend of higher gaze offset being associated with track position away from the outer edge (track position = 1.75) and toward the inner edge (track position = –1.75).

**Figure A6.**
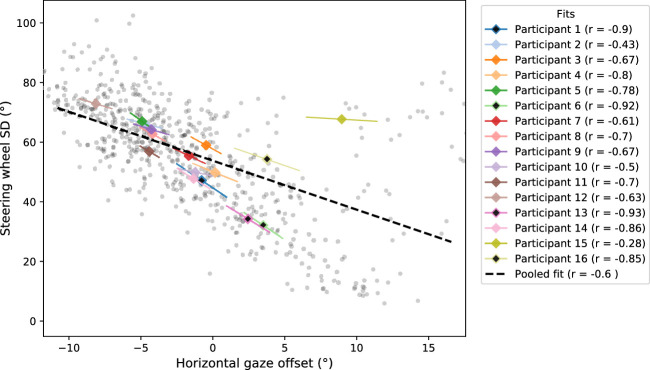
Scatterplot of steering wheel standard deviation as a function of horizontal gaze offset at the end of CURV-SEG = 9. Small gray dots indicate individual runs from all participants when WP_alt_ was missing during CURV-SEG = 9. Steering wheel deviations have been calculated from the 2 s following the inflection point. Colored lines indicate the slopes of participant-wise linear ordinary least squares fits (diamond the location of the participant mean). The pooled fit (dashed black line) has been calculated from the data of all participants. Participants 1, 6, 13, and 16 reported to have counted the WPs (see 3) and are marked with a black center within the diamond marker. All participants have a negative correlation with gaze offset, indicating less steering wheel deviation when gaze is more in the direction of WP_alt_. In addition to the within-participants effect, the participant means except for Participant 15 also appear to mostly follow the general trend of higher gaze offset being associated with lower variance of the steering wheel position.

More precisely, we calculated the horizontal gaze offset from the final frame of CURV-SEG = 9 and then calculated the mean track position from the following 2 s (i.e., CURV-SEG = 1 and CURV-SEG = 2) after the inflection point. With positive track positions indicating a position that was more toward the outer edge of the track and positive offset indicating a gaze position closer to WP_alt_ than WP_const_, every participant had a negative Pearson's correlation coefficient between gaze offset and track position (*t* test to test difference from 0, *p* < 0.0001) with a mean correlation (from Fisher-transformed participant correlations) of –0.70. The full trajectories in regards to gaze offset and both track position and x,y-coordinates can be seen in Figure 6. A scatterplot of the calculated mean track positions is visualized in Figure 7; otherwise, identical analysis but with steering wheel deviation as the dependent variable instead of mean track position is displayed in Figure A6.

### Participant strategies

At the end of the experiment, each participant answered a quick survey that checked whether they had used any specific strategies to predict when the direction of the track changed. Out of 16 participants, 4 (Participants 1, 6, 13, 16) mentioned that they had (at least at some point during the experiment) attempted to count waypoints to predict when the direction of the curve would change. However, when asked to estimate how many waypoint locations (i.e., the sum of visible and missing waypoints) there were there on a single constant radius curve, 6 participants (Participants 1, 6, 10, 13, 14, 16 in the participant-wise figures) were able to answer correctly.

## Discussion

Tuhkanen et al. (2019) observed that people shift their gaze toward the future path (marked by a series of waypoints) even when the visual information specifying the future path was withheld. These anticipatory gaze shifts toward the future waypoint location in the world took into account variation in road position and heading, indicating that gaze behavior was genuinely predictive (rather than being explained by a low-level motor response). However, these findings were potentially limited by the highly regular constant-radius paths that were used since most waypoints were positioned in very similar parts of the visual field. In the present study, we investigated whether the same anticipatory gaze behavior would occur when there were predictable—but visually withheld—changes in path direction. Participants steered through a series of S-bends (with an “inflection” point at the end of each bend), and the analyses confirmed previous findings: Gaze was directed to the predictable location of future waypoints, even when that waypoint was not visible.

Because of the alternating direction of bends, the location of the next waypoint varied according to how “deep” into each bend the driver had traveled when the waypoint was withheld. The first waypoint after bend inflection appeared in a markedly different location in the visual field compared to the preceding waypoints. If participants could determine how deep into each bend they had traveled (either through a spatial or temporal estimate or some combination thereof), they should be able to anticipate the bend change and shift gaze accordingly to predict the future path. Due to the sparse visual environment, any estimate should be uncertain (probabilistic), and so the gaze sampling from the “alternative” waypoint location should increase as the inflection point was approached.

Our analysis confirmed that gaze behavior did indeed change depending on how deep into each bend participants traveled. When visual cues to the future path were absent, participants’ gaze shifted more toward the alternative waypoint location the further they had traveled along the current bend. When waypoints along the future path were all visible, no such shift in gaze behavior was apparent (until the visible waypoint actually appeared at the alternative bend location). The observed gaze behavior provides evidence for greater visual anticipation than previously reported: both reflecting upcoming directional changes in steering, as well as increasing uncertainty about the likely direction of the future path.

It might be tempting to discount these changes in gaze behavior as being relatively “cheap”: Eye movements are low effort to produce and are low cost (in both time and energy) to change again in the future. By this logic, wider dispersal of gaze could simply be considered as some general search strategy when there is uncertainty without a clear relationship to the planned trajectory of the future path. But previous research has demonstrated that gaze and steering behaviors are tightly coupled (Land & Lee, 1994; Wilkie & Wann, 2003b; Chattington et al., 2007; Robertshaw & Wilkie, 2008; Kountouriotis et al., 2012) and, theoretically, may be derived from a common underlying representation (Land & Furneaux, 1997; Lappi & Mole, 2018; cf. also Tatler & Land, 2011). So, if gaze shifts were genuinely predictive of the expected path/planned trajectory, we should also observe shifts in steering to accompany the gaze shifts elicited when the bend inflection point was approached. Correlational analyses confirmed that steering did indeed shift in the direction of gaze (Figure 7), as would be expected if prediction was being used to direct gaze and inform steering control (Figure 6). While it is not possible to determine the direction(s) of causality between gaze and steering using these correlations, the directional links between gaze and steering have been investigated in previous experiments (Wilkie & Wann, 2003b; Kountouriotis et al., 2013; Mole et al., 2016).

We find it very difficult to explain the observed behaviors without recourse to some kind of internal representation of the future path. There appears to be no pure online strategy or even a stored (ballistic) motor program that could produce the observed results. While relying upon an internal representation might invoke the idea of a “high-fidelity world model,” it is also possible that a more or less “weak representation” could suffice (Zhao & Warren, 2015). The question here becomes whether gaze shifts toward the alternative waypoint were supported by a constantly updated (dynamic) estimate of some features of the path geometry, and where on the path they were currently located, or by some cognitive heuristic and/or (static) “spatial memory.” The participants might, for example, have been estimating time or distance or kept count of the waypoints in each curve.

Further computational modeling and experimental efforts should be made to tease apart these alternatives. But within our data, it does seem that there were individual differences in the approach to the task. Some participants appear to explicitly estimate how many waypoint locations each bend consisted of and kept a count of them to keep track of their location. With this cognitive strategy, a choice could then be made between producing a saccade “in the direction of rotation” or “in the opposite direction of rotation.” This could have been based on the display frame of reference or with reference to the actual future path in the world (“after x number of waypoint locations, the bend curvature will change”). It should be noted that this approach is not trivial since it requires the participants to count missing WPs as well as visible ones. While four participants (P1, P6, P13 and P16) explicitly reported attempting to count path features (waypoints/waypoint locations/curve segments—the exact features could not be distinguished based on the verbal reports), others may have done so without disclosing or explicitly realizing it. Regardless of whether the use of this cognitive heuristic would qualify as a “genuine” representation of the path geometry or not, it is certainly not a pure online strategy. And, for all the flaws of self-report methods, such an explicit strategy should have been straightforward for participants to report. Actually, a wide variety of strategies/approaches were reported, most of which were not straightforward to interpret (i.e., they don't simply map onto a counting waypoints rule), suggesting they were largely implicit. The lack of a single approach itself may be evidence that a form of model-based control was invoked since online control strategies should be highly consistent and (stereotypically at least) rely upon very few perceptually available signals. Alternatively, it could simply highlight a dissociation between the participants’ explicit understanding (what they think they are doing) and their control strategy (what they are actually doing) that has been observed previously (Mole et al., 2018).

Regardless of the exact strategies used, it is also apparent that participant performance was not perfectly accurate. A perfectly precise prediction should lead to identical gaze behavior irrespective of whether the waypoint was visible or not (i.e., minimal gaze fixations toward the alternative waypoint except at the bend inflection point). Instead, as participants traveled more deeply into each bend, we observed a gradual increase in sampling from the region around the alternative waypoint; the positive correlation between alternative waypoint sampling and curve segments persisted even if the last segment before the inflection point was excluded from analysis (this did not happen in the visible condition). This pattern might be explained by a gradual increase in uncertainty over whether the bend is about to change direction the further/longer one has traveled along the current constant-radius curve.

Consistent with the individual differences in self-reported strategies, it was also apparent that the gaze behaviors adopted by participants were not entirely uniform. When looking at the participant-wise figures (Figures A2 and A3), it is clear that the amount of anticipatory gaze behavior varied between participants, and the gradual increase in looking toward the alternative waypoint was not apparent for all participants. For example, Participant 15 frequently generated saccades toward the alternative waypoint but did so throughout the constant-radius curve; Participant 6 displayed almost identical behavior in the case of missing and visible waypoints (i.e., a sudden shift toward the alternative waypoint right before the infliction point) consistent with having a very precise estimate of their position on the bend; and among Participants 2, 5, 9, 11, and 12, the amount of anticipatory gaze behavior relative to the alternative waypoint was small at best. Regardless, for all participants, an increase in gaze position toward the alternative waypoint before the inflection point correlated with a track position closer to the inner edge (Figure 7) and less variance in steering (Figure A6) after the inflection point, suggesting that the anticipation of the alternative waypoint facilitated successful steering control.

### Conclusions

There is a long and ongoing theoretical debate about how an active moving animal anticipates future action requirements and adjusts their ongoing behavior accordingly. Anticipatory behaviors in humans can be observed in almost all skilled-action contexts, be it the timing of a ball catch or driving along a winding road at speed. However, successful anticipatory actions could result from fundamentally different processes: Explanations for anticipatory behavior range from model-free control to approaches that base action choice on internal representations. Model-free control relies upon a controller that is coupled directly to the information that is available to the organism and emerges from the animal's interaction with the environment (Zhao & Warren, 2015; Gibson, 1986) or on motor programs that run their course in a ballistic manner (the environmental information merely specifies when the motor program is launched and its parameters). Other forms of control are model based, meaning that the organism maintains some kind of structured internal representation about the environment (i.e., a memory structure that contains more than just a set of stored values of controller parameters; see Zhao & Warren, 2015; Bubić et al., 2010). If the organism further maintains an internal representation of the environment's dynamics and uses this to update its model, the representations are said to be generative or predictive.

For the field to progress, it is important that any underlying representations are probed by critical experiments complemented with formal modeling. In the present study, we demonstrate that when steering through a series of repeating S-bends, participants anticipate changes in track curvature with their gaze, even when visual cues of changes are withheld. This anticipation reflected the increased uncertainty over the future path as the inflection point of the S-bend was approached, strongly suggesting that steering control is informed by some form of internal representation. Prediction appears to play a critical role when it comes to explaining the observed gaze and steering behaviors.

## Figures and Tables

**Figure A4.**
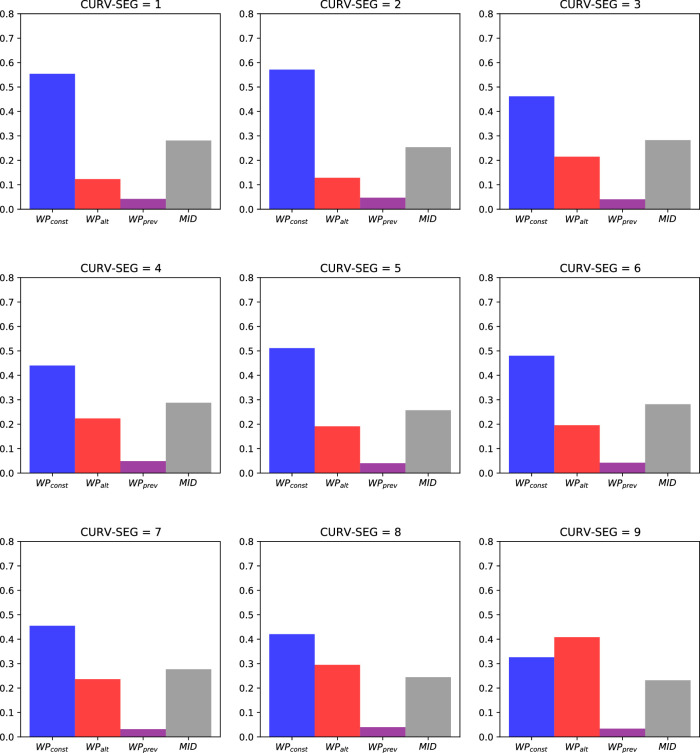
The ratio of saccades classified to each possible class in the MISS condition for each CURV-SEG. Data have been pooled from all participants and test trials.

**Figure A5.**
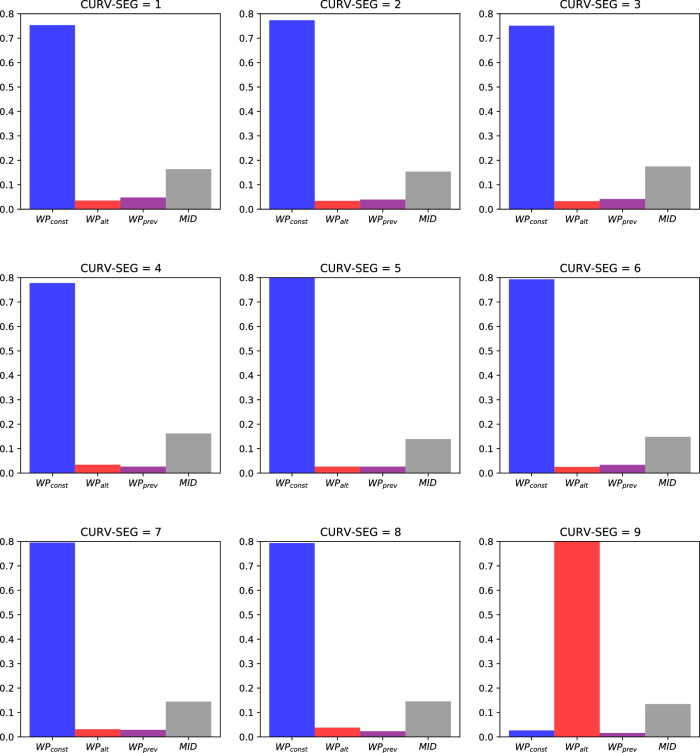
The ratio of saccades classified to each possible class in the VIS condition for each CURV-SEG. Data have been pooled from all participants and test trials.

**Figure A7.**
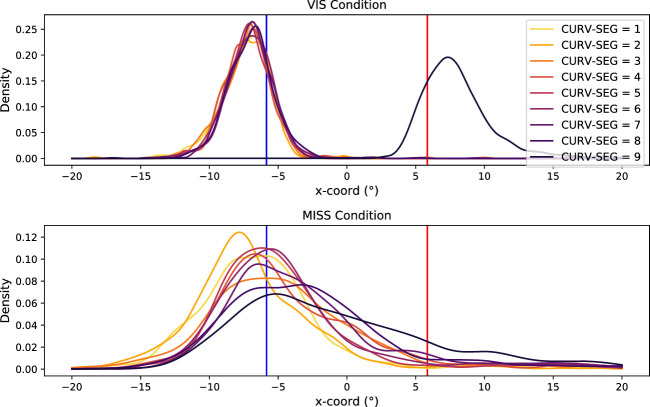
Gaussian density estimates of the horizontal gaze distribution in the last frame before the end of a CURV-SEG in both the VIS (top panel) and MISS condition (bottom panel). The vertical red line indicates the median position of WP_alt_ and the vertical blue line the median position of WP_const_. Gaze positions have been referenced to the middle point between WP_alt_ and WP_const_. Note the widening of the distribution in the direction of the WP_alt_ in the later curve segments. All data were pooled for the analysis, and left-turning CURV-SEGs were mirrored horizontally.

**Figure A8.**
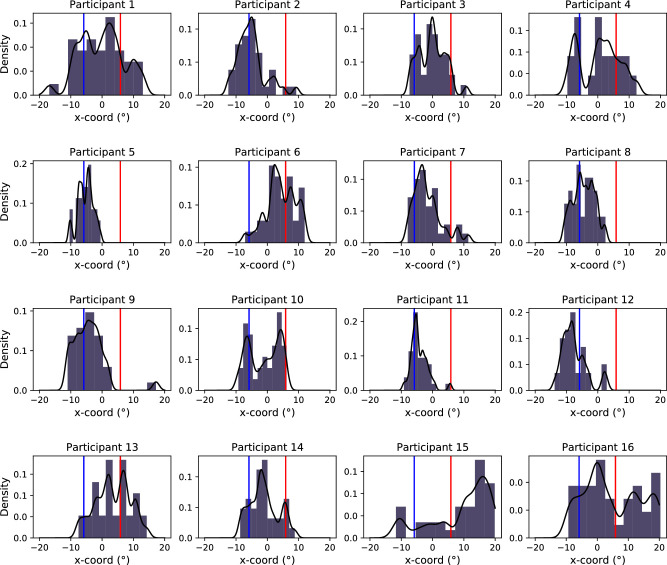
Participant-wise Gaussian density estimates of the horizontal gaze distribution in the last frame before the end of a CURV-SEG = 9 in the MISS condition. Gaze positions have been referenced to the middle point between WP_alt_ and WP_const_. The vertical red line indicates the median position of WP_alt_ and the vertical blue line the median position of WP_const_.

**Figure A9.**
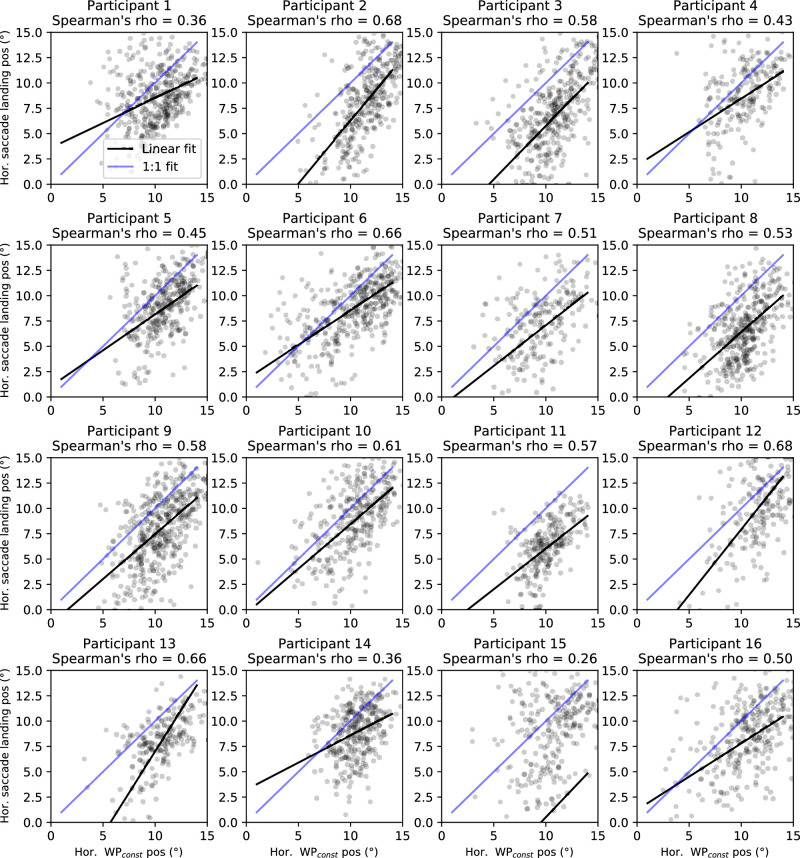
Horizontal saccade landing positions as a function of the position of WP_const_. All saccades during 1 ≤ CURV-SEG ≤ 8 in the MISS condition are included. All participants have a positive correlation. This suggests that the drivers did not target saccades to a fixed point on the screen but rather were at least somewhat adaptive to the prevailing circumstances regarding the position of the invisible WP. This replicates a comparable analysis in Tuhkanen et al. (2019).

**Figure A10.**
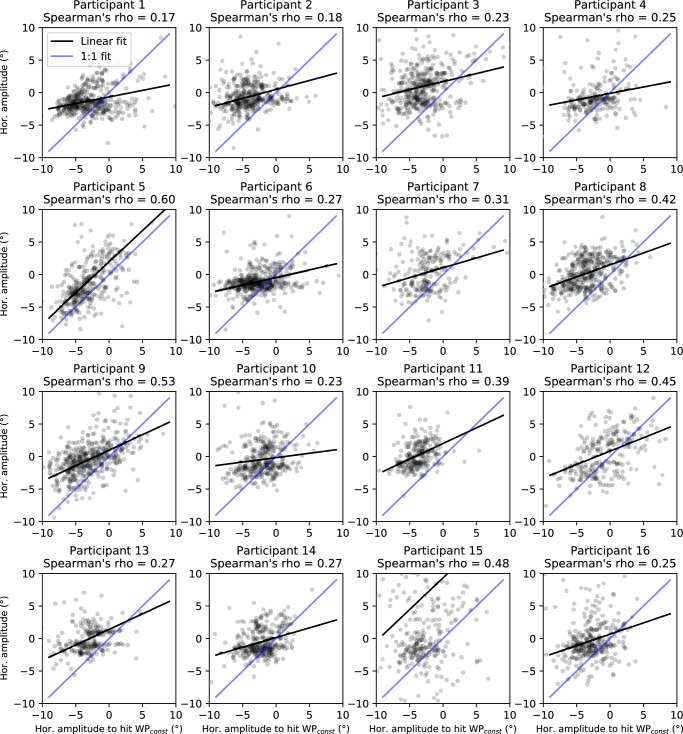
Horizontal saccade amplitudes as a function of what amplitude would be required to hit WP_const_. All saccades during 1 ≤ CURV-SEG ≤ 8 in the MISS condition are included. All participants have a positive (though generally small) correlation. This suggests that the participants did not perform saccades with fixed amplitudes but rather were (weakly) adaptive to either the location of the launch point and/or the position of the invisible WP. This replicates a comparable analysis in Tuhkanen et al. (2019).
